# The Prognostic Value of Lysine Acetylation Regulators in Hepatocellular Carcinoma

**DOI:** 10.3389/fmolb.2022.840412

**Published:** 2022-03-09

**Authors:** Liying Sun, Jian Zhang, Kai Wen, Shenglan Huang, Dan Li, Yongkang Xu, Jianbing Wu

**Affiliations:** ^1^ Department of Oncology, The Second Affiliated Hospital of Nanchang University, Nanchang, China; ^2^ Jiangxi Key Laboratory of Clinical and Translational Cancer Research, Second Affiliated Hospital of Nanchang University, Nanchang, China; ^3^ Department of Orthopedics, The Second Affiliated Hospital of Nanchang University, Nanchang, China; ^4^ Department of Hepatobiliary Surgery, Sun Yat-Sen Memorial Hospital, Sun Yat-Sen University, Guangzhou, China

**Keywords:** lysine acetylation regulators, hepatocellular carcinoma, prognostic signature, overall survival, nomogram

## Abstract

**Background:** Hepatocellular carcinoma (HCC) is a tumor with high morbidity and mortality worldwide. lysine acetylation regulators (LARs) dynamically regulate Lysine acetylation modification which plays an important regulatory role in cancer. Therefore, we aimed to explore the potential clinical prognostic value of LARs in HCC.

**Methods:** Differentially expressed LARs in normal liver and HCC tissues were obtained from The Cancer Genome Atlas (TCGA) and International Cancer Genome Consortium (ICGC) datasets. To identify genes with prognostic value and establish the risk characteristics of LARs, consensus clustering was employed. We used univariate Cox regression survival analysis and LASSO Cox regression based on LARs to determine the independent prognostic signature of HCC. CIBERSORT and Gene Set Enrichment Analysis (GSEA) were used to estimate immune infiltration and functional enrichment analysis respectively. The expression of LAR was detected by Real-time quantitative polymerase chain reaction (RT-qPCR). statistical analyses were conducted using SPSS and R software.

**Results:** In this study, the 33 LARs expression data and corresponding clinical information of HCC were obtained using TCGA and ICGC datasets. We found majority of the LARs were differentially expressed. Consensus cluster analysis was carried out based on the TCGA cohort, and three HCC subtypes (cluster 1, 2, and 3) were obtained. The LA3 subgroup had the worst clinical outcomes. Nine key LARs were identified to affect prognosis. The results showed that LARs signature has a strong independent prognostic value in HCC patients, whether in the training datasets or in the testing datasets. GSEA results showed that various tumor-related processes and pathways were abundant in the high-risk groups. RT-qPCR results showed that HAT1, HDAC1, HDAC2, HDAC4, and HDAC11 were highly expressed in HCC cells.

**Conclusion:** Our results suggest that LARs play critical roles in HCC and are helpful for individual prognosis monitoring and clinical decision-making of HCC.

## Introduction

Hepatocellular carcinoma (HCC) is one of the most common malignant tumors worldwide, ranking sixth in cancer incidence and fourth in mortality worldwide (Kulik and El-Serag, 2019; Huang et al., 2021). HCC risk factors include viral infections, obesity, nonalcoholic fatty liver disease, and high intake of aflatoxin (Caruso et al., 2021). Progress has been made in recent years regarding HCC, and the clinical prognosis of HCC has improved to a certain extent.(Fan, 2012). However, due to the high heterogeneity and high incidence of recurrence and metastasis, the overall prognosis with HCC remains unsatisfactory (Nault et al., 2020). Therefore, it is urgent to need to develop novel therapeutic targets for HCC patients, and act as effective predictive markers to evaluate HCC patient prognosis.

Cancer is a multifactorial disease that results from the interaction between genetic abnormalities and epigenetic changes. Epigenetic changes are an important sign of cancer progression (Baylin and Jones, 2016; Feinberg et al., 2016; Cavalli and Heard, 2019). Post-translational modification (PTM) is an important way to regulate protein function and affect cell behavior, and act as a signal marker in cancer cells (Chen et al., 2020; Figlia et al., 2020). Lysine acetylation is an important reversible and dynamic protein PTM that is exceedingly important for gene expression. It plays an important role in transcription factor activity, chromatin remodeling, and metabolic enzyme activity, and is related to tumorigenesis, tumor progression, and metastasis (Choudhary et al., 2014; Kaypee et al., 2016; Narita et al., 2019). Lysine acetylation is a reversible epigenome modification regulated by two clusters of opposing enzymes: lysine acetyltransferases (KATs) and histone deacetylases (HDACs) (Falkenberg and Johnstone, 2014; Sheikh and Akhtar, 2019). KATs are primarily divided into two types according to their cellular localization and acetylated chromatin histone ability: type a KATs and type b KATs. The main KAT families are the GCN5-related N-acetyltransferase (GNAT) (Bienvenut et al., 2020), p300/CBP (KAT3) (Lipinski et al., 2020)and MYST (MOZ, Ybf2, Sas2, and TIP60) families (Baell et al., 2018). Moreover, two other KAT families belong to the nuclear receptor family of transcription factor-related KATs and KATs. HDACs are roughly divided into classical HDACs, including type I (homologues of yeast Rpd3, including HDAC 1, 2, 3, and 8), II (homologues of yeast Hda1, including HDAC 4, 5, 6, 7, 9, 10), IV (HDAC 11), and NAD + -dependent III HDAC or sirtuins, similar to yeast Sir2 (Li et al., 2020). Although the molecular regulation mechanism of LARs in HCC has been discussed, there is a lack of comprehensive research (Chai et al., 2021). Therefore, we systematically studied the role of LARs in HCC in order to provide potential prognostic markers and therapeutic targets.

In this study, we grouped 368 HCC patients based on LARs and identified three subgroups with differences in prognosis. The risk score be calculated by the LASSO-Cox regression to establish a LARs signature. We also discuss the relationship between the LAR risk model and immunity. Finally, we performed experiments to verify the LARs mRNA expression. Our findings reveal the possible role of LARs in HCC, which is of substantial significance for accurate HCC treatment.

## Materials and Methods

### Data Acquisition

RNA-seq data for HCC samples were selected from the TCGA dataset (https://portal.gdc.cancer.gov/), and clinical data related to the patient were downloaded simultaneously. The study included 368 HCC and 50 normal liver tissue samples. The RNA-seq data of the verification set and the corresponding clinicopathological information were downloaded from ICGC dataset (https://dcc.icgc.org/), including 232 HCC samples and 202 normal samples adjacent to cancer. We obtained 33 LARs were obtained from previously published literature (Narita et al., 2019), of which 13 belonged to the acetyltransferase family, and 20 belonged to the deacetylase class. The 33 LARs information is provided in Supplementary Table S1.

### Analysis of the LARs Regulatory Factors in HCC

The differential expression of LARs in HCC and normal liver tissue samples was analyzed with the “limma” R package and visualized by heatmap. The protein-protein interactions (PPI) network between differentially expressed LARs were analyzed with the STRING website (http://string-db.org/). In addition, the correlation between these LARs was calculated using Pearson correlation network analysis.

### Consensus Clustering Analysis

In the TCGA cohort, the expression of 33 LARs were analyzed to determine the HCC subtype by the “ConsensusClusterPlus” R package (Wilkerson and Hayes, 2010). The method was based on the classical K-Means algorithm, with the Euclidean distance, iterated 50 times, and 80% of tumor samples were taken in each iteration. The number of clusters was set to 2–9, and we determine the best cluster number through the clustering score for the cumulative distribution function (CDF). According to different subgroups, the survival curve was drawn with survival R packet. Combined with clinical characteristics, heat maps were used to analyze the expression and distribution of LARs in different subgroups.

### Establishment and Verification of a Prognostic Risk Model

In the TCGA dataset, the survival information of HCC patients and the expression data of LARs were combined, and Cox regression analysis was performed to obtain the hazard ratio (HR) with its 95% confidence interval (CI) for each LARs. According to HR to identify protective genes (HR < 1) and dangerous genes (HR > 1), LARs with *p* < 0.05, were selected for the next analysis. According to the LARs related to prognosis, nine risk genes were identified using the LASSO algorithm, and the risk coefficient of each gene was obtained. We constructed a risk-scoring equation based on LAR expression:

$$Risk\;score=\overset{n}{\underset{i=1}{\sum }}\left(Coef_{i}*x_{i}\right)$$

where $Coef_{i}\;$ refers to the risk coefficient of the gene and $x_{i}$ is the expression value of the gene.

Taking the median risk score as the cutoff value, HCC patients were classified into high-risk and low-risk group. Using the “survival” R package to perform Kaplan–Meier survival curve analysis, we drew a risk curve from low to high patient risk values, and the package “timeROC” was used to depict the ROC curve, calculate the area under the curve (AUC), and evaluate the accuracy, the sensitivity and the specificity of the model. Principal component analysis (PCA) and t-distribution stochastic neighbor embedding (t-SNE) methods were used to study the distribution of different populations by using R packages stats and Rtsne.

### Construction and Verification of Predictive Nomogram

Clinicopathological characteristics and risk scores were integrated with survival data to obtain independent prognostic analysis input files, and The R package “survival” was used to perform univariate Cox and multivariate Cox regression analyses on the input files to evaluate the role of predictive model risk scores in prognostic prediction. The results were a forest map for visualization. Then, we used the “rms” R package to construct these independent prognostic factors, and constructed 1-, 3-, and 5-years forecast nomograms and corresponding calibration charts. The concordance index (C-index) was applied to evaluate the performance of the nomogram, and the calibration curve of the nomogram was used to evaluate the consistency between actual and predicted value.

### Immune Infiltration Analysis

Based on the TCGA database, the “CIBERSORT” R package was used to calculate the infiltration proportions of 22 immune cells in each HCC patient and displayed with a barplot, heatmap, and violin chart. The correlation between the expression and distribution of 22 infiltrating immune cells in the two groups was analyzed, and the related heat map was drawn. Red denotes a positive correlation, blue indicates a negative correlation.

### Gene Set Enrichment Analysis

Gene expression data were analyzed by Gene Set Enrichment Analysis (GSEA) software (http://www.gsea-msigdb.org/gsea/index.jsp). The related pathways and molecular mechanisms of HCC patients were evaluated using the “Hallmark” and the “KEGG” gene sets. The minimum and maximum gene set sizes were 10 and 500, respectively. The nominal (NOM) *p*-value < 0.05 and false discovery ratio (FDR) q value < 0.25 were considered statistically significant.

### Cell Culture

Human hepatic cell line, THLE-2 was purchased from the American Type Culture Collection (MA, USA), and HCC cell line (MHCC-97H, HepG2, LM3) was purchased from the National Collection of Authenticated Cell Cultures (Shanghai, China). The cell lines were cultured in Dulbecco’s modified Eagle medium (Gibco, TX, USA), supplemented with 10% fetal bovine serum (Gibco) in a humidified incubator (37°C, 5% CO_2_).

### Reverse-Transcription Quantitative PCR

Total RNA was extracted from cells using TRIzol reagent (Invitrogen, MA, USA). cDNA was synthesized using the PrimeScript RT reagent kit (Takara, Shiga, Japan) following the manufacturer’s instructions. The samples were amplified by qPCR using SYBR Green qPCR Master Mix (Thermo Fisher Scientific, MA, USA). The primer sequence information is provided in Supplementary Table S2.

### Statistical Analysis

All statistical analyses were conducted using R. 4.0.4 (https://www.r- project. org/) and SPSS Statistics software (version 25, https://www.ibm.com/products/software). The student’s t-test was used to compare the differences between the two groups. A value of *p* < 0.05 indicated that a statistically significant difference.

## Results

### Expression Characteristics and Interactions of LARs

**FIGURE 1:**
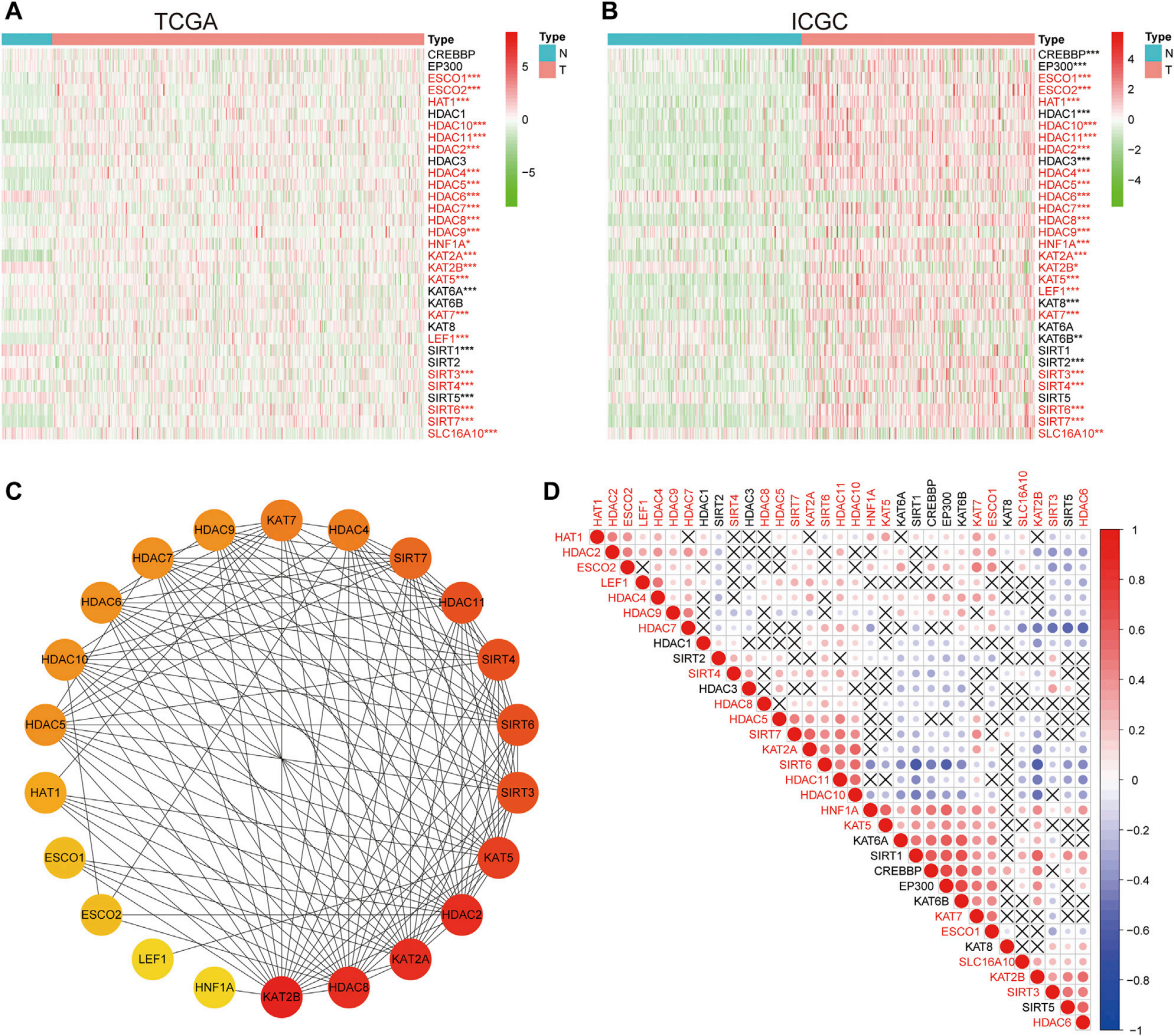
The expression characteristics and correlation of LARs in hepatocellular carcinoma (HCC). **(A)** Heatmaps present the overall expression of LARs in HCC tissues and normal liver tissues from The Cancer Genome Atlas (TCGA). The red mark indicates that the gene is differentially expressed in the two data sets. **(B)** Heatmaps presented the overall expression of LARs in HCC tissues and normal liver tissues from the International Cancer Genome Consortium (ICGC). **(C)** STRING online tool was used to analyze the interaction of LARs regulatory factors. **(D)** Spearman correlation analysis of 33 regulatory genes in the HCC cohort. *p < 0.05, **p < 0.01, ***p < 0.001.

The expression characteristics of 33 LARs were analyzed using TCGA and ICGC datasets. Compared with normal liver tissues, 23 same LARs were abnormally expressed in HCC in the ICGC and TCGA datasets (Figures 1A,B) Genes marked in red indicate that they were differentially expressed in the two databases. Considering the similarity of biological functions of LARs, we analyzed the interaction and correlation between 33 LARs differentially expressed in the TCGA dataset. A PPI network analysis showed that these LARs frequently interact with each other, and HDACs and KATs have a particularly high interaction with other LARs (Figure 1C). Figure 1D shows the correlation of 33 LARs in HCC. We found that there was a significant correlation between the gene expression patterns of LARs in the same functional category, and there was a high correlation between acetyltransferase and deacetylase. In correlation analysis, there was a high correlation among KAT2A, HDAC10, SIRT6 and SIRT7, while SIRT6 was negatively correlated with the expression of KAT2B, HDAC6 and HDAC7 in HCC.

### Identification of Hepatoma Subsets by Consensus Clustering

**FIGURE 2:**
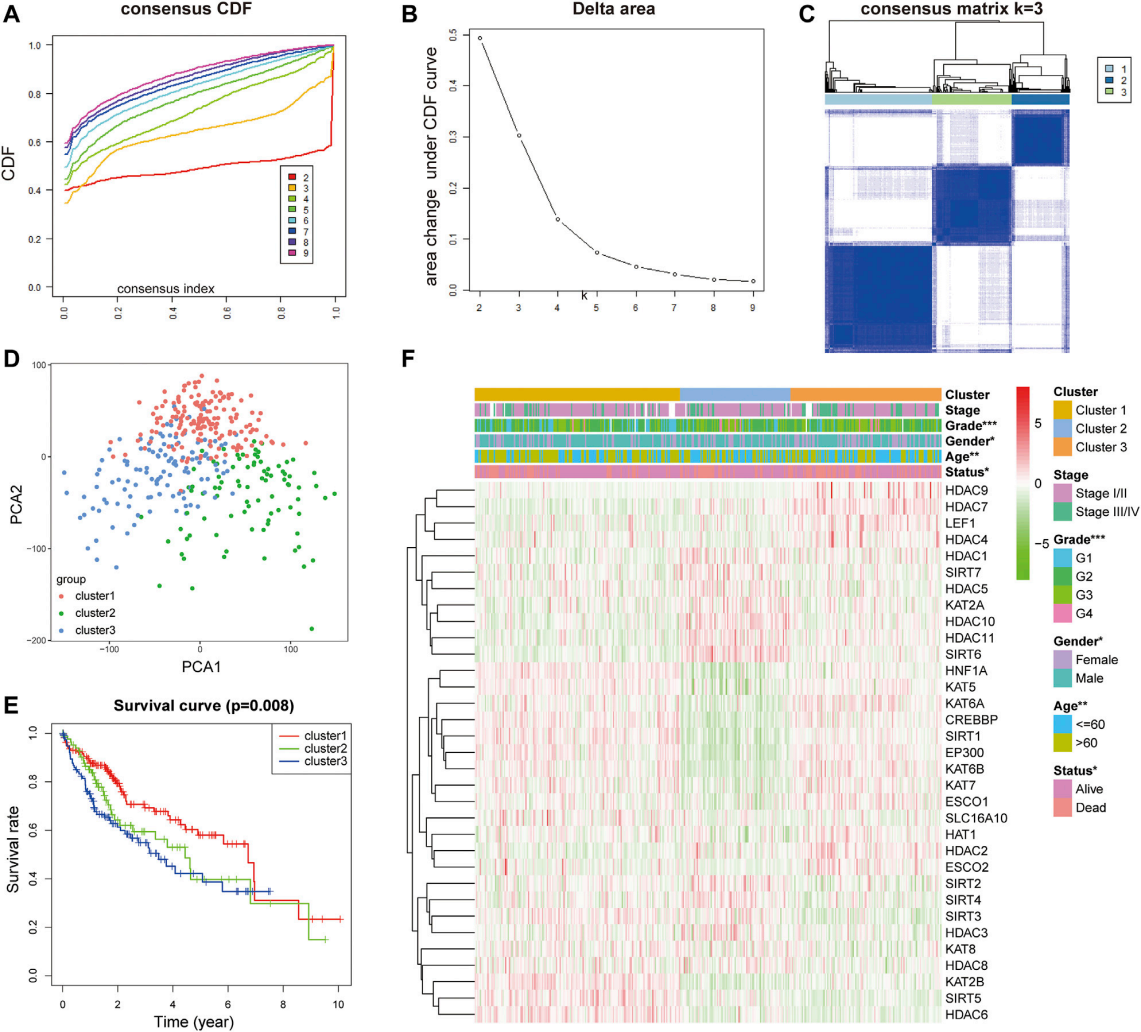
**(A)** Consensus clustering cumulative distribution function (CDF) for k = 2 to 9. **(B)** Relative change in area under the CDF curve for k = 2 to 9. **(C)** Consensus clustering matrix for k = 3. **(D)** PCA of the total RNA expression profile in TCGA dataset. **(E)** Kaplan–Meier survival curves for HCC in the three subgroups defined by the consensus expression of 33 LARs. **(F)** The different expression levels of LARs and clinicopathological feature contributions.

In order to establish the prognostic characteristics based on LARs, Pearson correlation coefficient analysis was carried out. Consensus clustering analysis was used to divide 368 samples into k groups (k = 2–9) (Figures 2A,B). According to the cumulative distribution function (CDF) value, k = 3 is the best number of clusters for dividing HCC queues, that is, cluster 1, cluster 2 and cluster 3 (Figure 2C). In order to understand the differences among the three subgroups in more detail, we used principal component analysis (PCA) to evaluate the classification of mRNA expression profiles, and the analysis showed that there were significant differences among the three subgroups (Figure 2D). In addition, the results of survival analysis showed that there was a great difference in OS time among the three clusters, and the OS of patients with cluster 3 was significantly lower than that of cluster 1 (Figure 2E). Subsequently, we evaluated the relationship between clusters and clinicopathological features (Figure 2F). There are differences in pathological characteristics such as grade, age, sex, and status among the three groups, and there are also differences in expression levels of LARs between the three clusters.

### Building a LARs Signature Using LASSO Cox Regression

**FIGURE 3:**
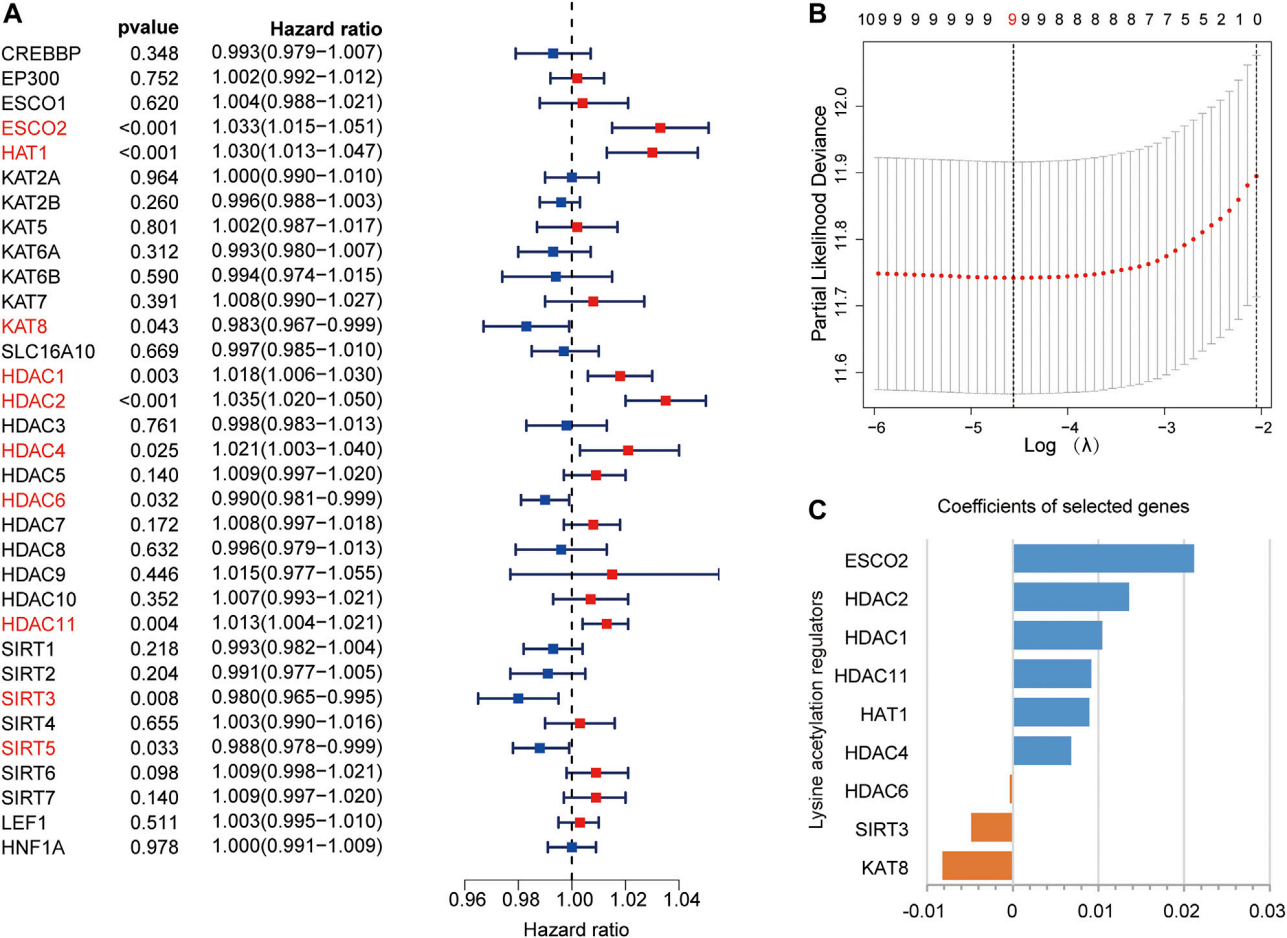
**(A)** Univariate Cox regression analysis was used to calculate the hazard ratios (HRs), 95% confidence intervals, and *p*-values for screening the prognostic LARs. **(B)**, **(C)** LASSO regression was performed to calculate the minimum criteria **(B)** and coefficients **(C)**.

In the TCGA dataset, we performed univariate Cox regression analysis to explore the prognostic values of LARs. We found that 10 LARs were significantly associated with OS in HCC patients (Figure 3A). Among the 10 genes, ESCO2, HAT1, HDAC1, HDAC2, HDAC4, and HDAC11 are risk factors for HCC, with a risk ratio >1, while KAT8, HDAC6, SIRT3, and SIRT5 are protective factors, with a risk ratio <1. LASSO cox regression analysis was then used to build a prognostic model from the 10 LARs for HCC patients in the TCGA dataset. Based on the minimum standard, the prognostic features based on 9 LARs are successfully developed (Figure 3B, Supplementary Figure S1A). Nine LARs coefficients were visualized from high to low using the LASSO algorithm, as shown in Figure 3C. Detailed information is provided in Supplementary Table S3.

### Evaluation and Validating of the Prognostic Model in TCGA Dataset

**FIGURE 4:**
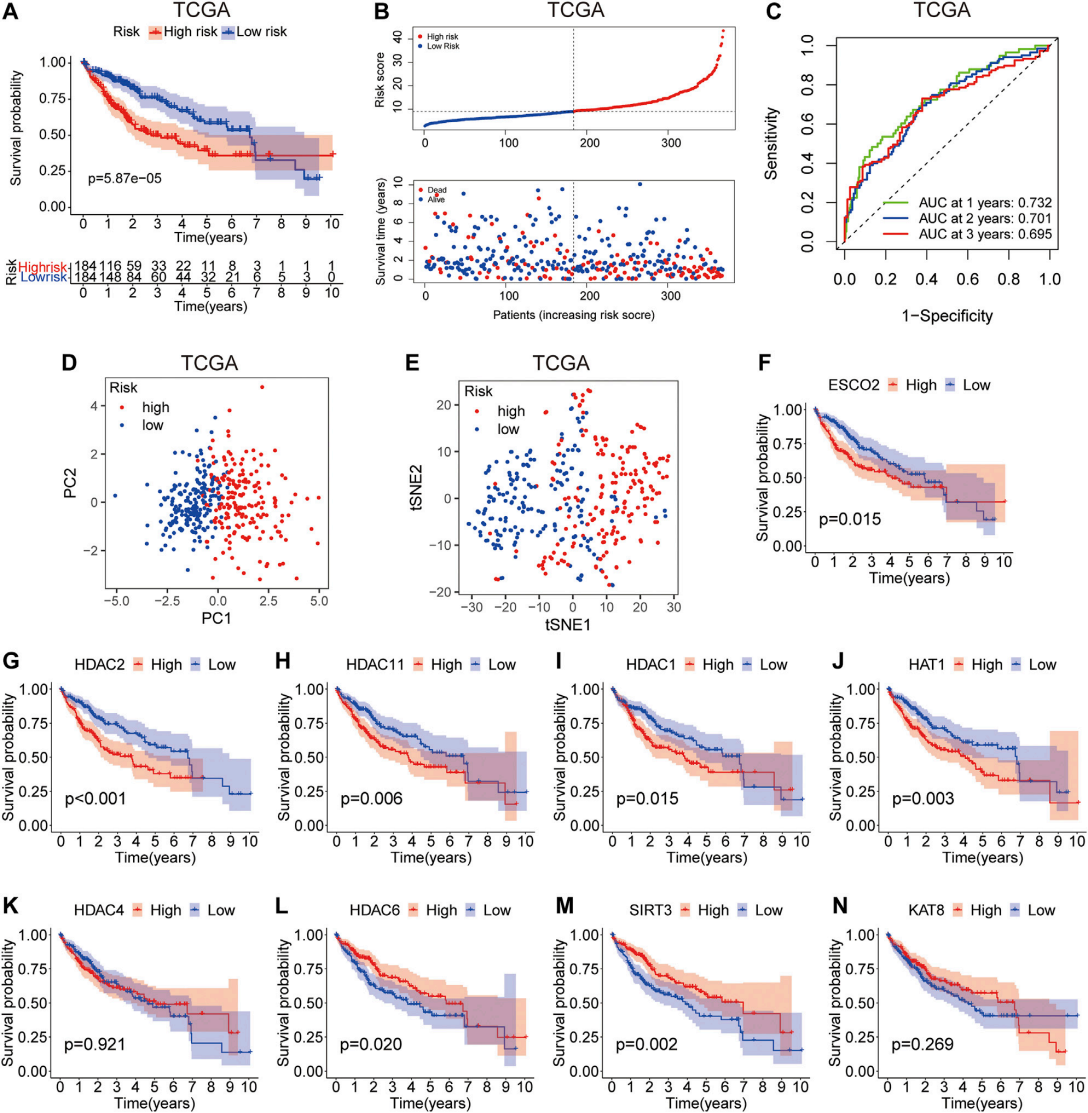
Prognostic value of the risk model in TCGA cohort. **(A)** Survival analysis of patients in the high-risk and low-risk groups based on the prediction risk score formula. **(B)** The median value of risk scores with survival and statuses of HCC patients depends on the risk model in the TCGA cohort and the distribution of risk scores with survival and statuses of HCC patients depends on the risk model in the TCGA cohort. **(C)** 1-, 2-, and 3-years receiver operating characteristics (ROC) curves of the risk model for assessing the prognostic performance of the gene signature in the TCGA cohort. **(D)** Principal component analysis of HCC patients in TCGA cohort. **(E)** t-distributed stochastic neighbor embedding (t-SNE) analysis of HCC patients in TCGA cohort. **(F–N)** Kaplan–Meier survival analysis of the association between mRNA expression of LARs and OS in HCC patients. The HCC sample information was derived from TCGA databases.

According to the risk score, HCC patients in the training group were divided into high-risk group and low-risk group. Survival analysis showed that patients in the high-risk group had worse survival compared with those in the low-risk group (Figure 4A). The increase in LARs-related gene expression and mortality in HCC patients were parallel to the increase in risk score (Figure 4B). The AUC values of 1-year, 2-years and 3-years calculated by TCGA dataset are 0.732, 0.701 and 0.695 respectively (Figure 4C), indicating that our LARs prognostic characteristics have a good predictive ability. PCA and t-SNE analysis revealed that distribution patterns of patients were significantly different in the different risk groups (Figures 4D,E). Then, Kaplan-Meier survival analysis was performed to investigate the prognostic value of a single LAR in HCC patients. The results showed that the expression of ESCO2, HDAC2, HDAC11, HDAC1, HAT1, HDAC6, and SIRT3 were found to be linked to the prognosis of HCC (Figures 4F–N). To evaluate the predictive value of LARs signature obtained from the training set, we used the ICGC database as external verification dataset. Using the same formula, the risk scores of the patients in the validation set were calculated and grouped. As shown in Supplementary Figures S2A,B, the survival time of patients in the high-risk group was significantly shorter than that in the low-risk group (*p* < 0.001). Moreover, the verification results showed that the AUC expression of LAR-related mRNA was 0.752, 0.656, and 0.685 at 1, 2, and 3 years, respectively (Supplementary Figure S2C). The PCA and t-SNE analysis successfully separated and confirmed two patient subgroups (Supplementary Figures S2D–E). Kaplan-Meier survival curve revealed that patients with high expression of ESCO2, HDAC2, HDAC11, HAT1 and HDAC6 had poorer OS expression than patients with low expression. (Supplementary Figures S2F–N), which is consistent with results from training set. These results revealed that the LARs signature is excellent in predicting the prognosis of HCC in the validation set.

### Independent Prognostic Value of LARs Signature

**FIGURE 5:**
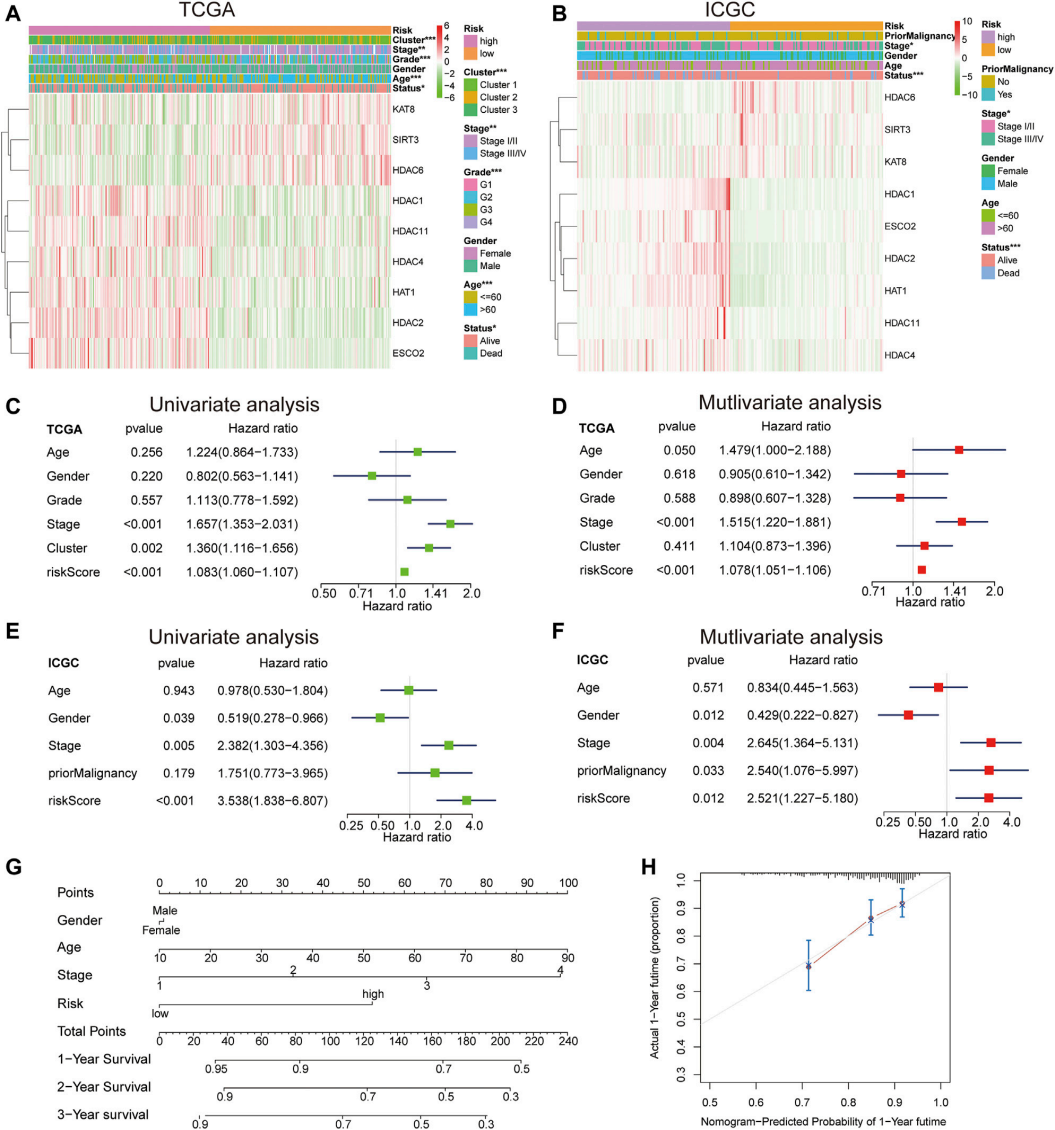
**(A)** The differential expression levels of the included 9 LARs and the distributions of clinicopathological characteristics were compared between low- and high-risk subgroups in the TCGA cohort. **(B)** The differential expression levels of the included 9 LARs and the distributions of clinicopathological characteristics were compared between low- and high-risk subgroups in the ICGC cohort. **(C)**, **(D)** Univariate **(C)** and multivariate **(D)** Cox regression analyses of the OS and clinicopathological features of patients from TCGA datasets. **(E)**, **(F)** Univariate **(E)** and multivariate **(F)** Cox regression analyses of the OS and clinicopathological features of patients from ICGC datasets. **(G)** A line chart was established to predict the 1-, 3-, and 5-years survival rates of HCC patients. **(H)** The 1-year alignment diagram calibration curve of the entire TCGA queue.

According to the heat map of risk and clinical correlation, the high-risk score was positively correlated with tumor classification, stage, grade, age, and status in the TCGA dataset (Figure 5A). In the ICGC dataset, the high-risk group was positively correlated with stage and status (Figure 5B). According to the risk and clinical correlation heat map of TCGA and ICGC datasets, HDAC1, HDAC2, HDAC4, HDAC11, HAT1, and ESCO2 were suggested to be high-risk LARs. In TCGA cohort, according to univariate Cox regression analysis, staging (HR = 1.657, 95% CI, 1.353–2.031, *p* < 0.001) and risk score (HR = 1.083, 95% CI, 1.060–1.107, *p* < 0.001) were significantly correlated with OS (Figure 5C). Multivariate analysis by Cox regression showed that, after correcting for other confounding factors, staging (HR = 1.515, 95% CI, 1.220–1.881, *p* < 0.001) and risk score (HR = 1.078, 95% CI, 1.051–1.106, *p* < 0.001) were still statistically significant (Figure 5D). The results were validated using the ICGC queue (Figures 5E,F). Based on multivariate analysis by Cox regression, sex, age, stage, and risk scores were introduced into the line chart to quantitatively predict the OS of HCC patients (Figure 5G). The calibration curves of the 1-, 3-, and 5-years line diagrams were very close to the best prediction curve, and the predicted OS rate and the actual observation value were highly consistent. (Figure 5H, Supplementary Figures S1B–F).

### Correlation Between LARs-Related Signature and Immune Infiltration

**FIGURE 6:**
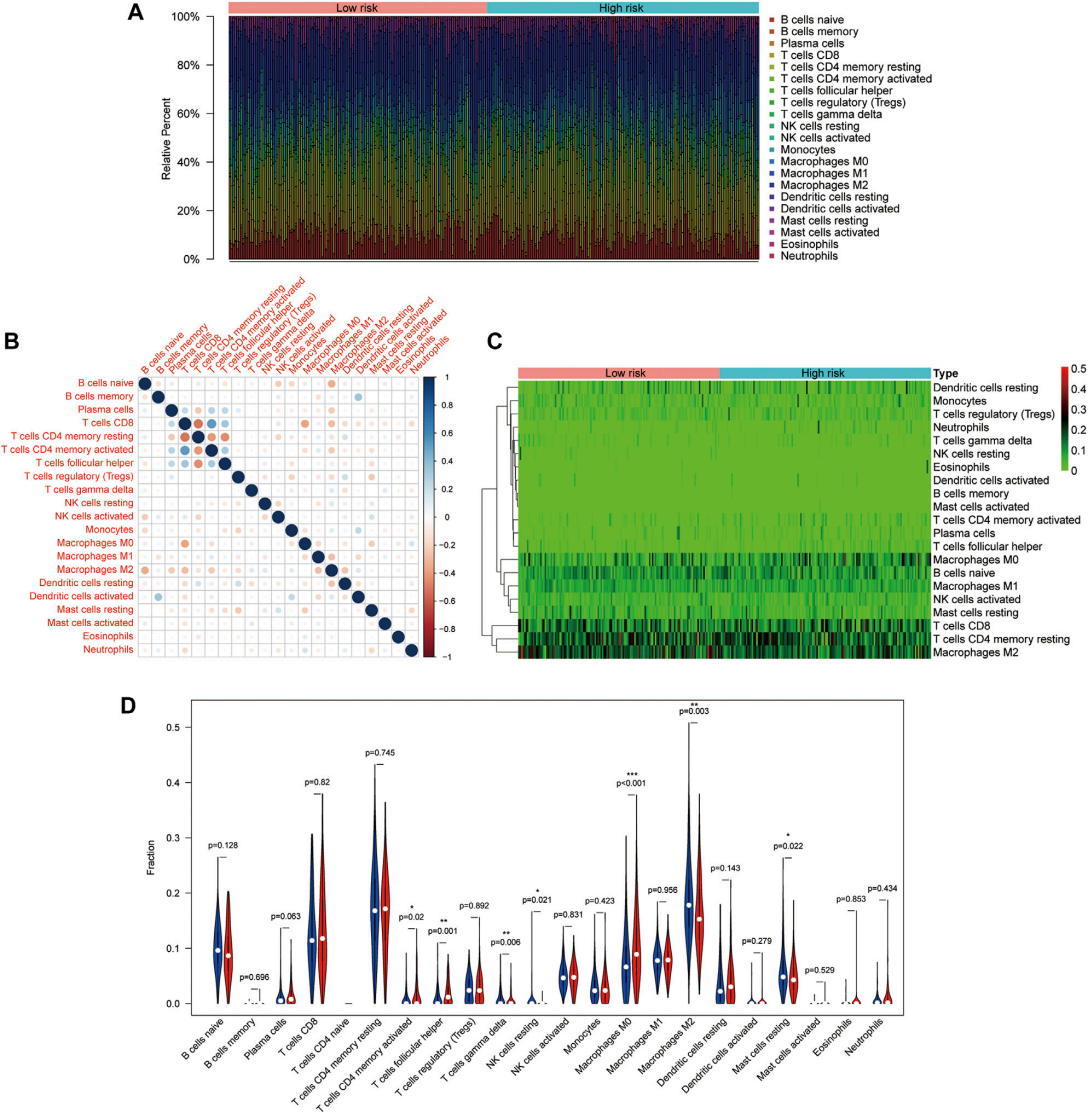
Distribution and visualization of immune cell infiltration in HCC patients. **(A)** The histogram shows 21 specific immune components represented by different colors in each sample. **(B)** The correlation matrix of the proportion of 21 types of immune cells. **(C)** DEIRGs heat map of patients with high and low tumor mutational load (TMB). **(D)** Violin pictures with different degrees of immune cell infiltration in patients with high and low TMB. Blue and red represent low- and high-risk samples, respectively.

To further immune infiltration analysis, we employed CIBERSORT to calculate the infiltration abundance of 22 immune cell types between high-and low-risk HCC samples in the TCGA dataset. The infiltration ratio of immune cells was shown in Figure 6A. We also assessed the association between 22 types of immune cells, and the heat map revealed that the proportions of different subpopulations of tumor-infiltrating immune cells were weakly to moderately correlated (Figure 6B). Next, we used the heat map (Figure 6C) and the violin map (Figure 6D) to reveal possible differences in immune cell expression between groups. The results showed that memory activated CD4^+^ T cells and macrophages M0 were higher expressed in the high-risk group, while T cells gamma delta, NK cells regulatory, Macrophages M2, T cells follicular helper, and Resting mast cells were more infiltrated in the low-risk group.

### Gene Set Enrichment Analysis

**FIGURE 7:**
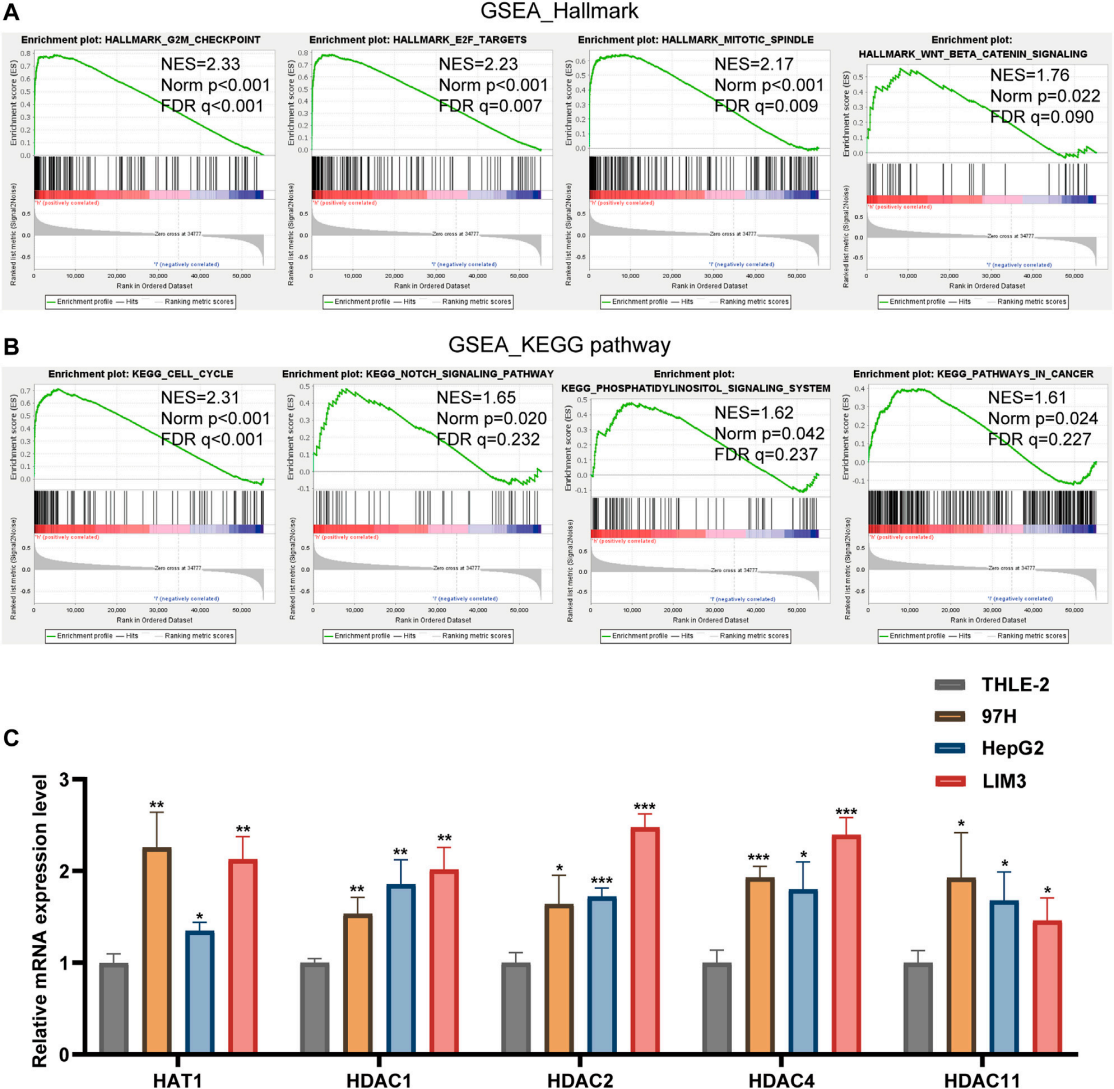
**(A,B)** Gene Set Enrichment Analysis was conducted to predict the potential functions and pathways regulated by LARs based on TCGA datasets. **(C)** The RT-qPCR results of the 5 LARs genes was evaluated using the 2^-ΔΔCT^ method. *p < 0.05, **p < 0.01 and ***p < 0.001.

GSEA was used to study tumor markers and signal pathways in patients with low-risk and high-risk HCC in TCGA dataset. We determined that four tumor characteristics, namely E2F targets, G2/M checkpoint, mitotic spindle and Wnt/β-catenin signaling were significantly enriched in high-risk HCC patients (Figure 7A). GSEA-based KEGG pathway analysis confirmed that cell cycle, notch signaling pathway, pathways in cancer and phosphatidylinositol signaling system were enriched in the high-risk subgroup (Figure 7B).

### Validation of LARs Expression Levels

We used RT-qPCR to verify the expression level of these prognosis-related LARs genes.

Our results showed that compared with the HCC cell line (THLE-2), the expression of HAT1, HDAC1, HDAC2, HDAC4 and HDAC11 were up-regulated in the HCC cell line (97H, HepG2, LIM3) (Figure 7C).

## Discussion

HCC is a serious malignant tumor worldwide. Furthermore, the incidence of HCC has continued to increase (Yang J. D. et al., 2019). Considering the huge heterogeneity of HCC, there is an urgent need to identify new prognostic biomarkers and establish a more accurate prognostic model. Lysine acetylation is a common cellular protein modification that regulates several cellular processes and participates in tumorigenesis and metastasis (Sabari et al., 2017). Related HCC studies have reported that LARs play an important role in HCC (Chai et al., 2021). Therefore, LARs may have considerable potential as biomarkers to predict HCC patient prognosis.

In this study, We found that the mRNA expression levels of majority of the evaluated LARS were closely related to the clinicopathological characteristics of HCC. Through the differential expression analysis of 852 genes, we screened 33 genes as potential prognostic factors to construct a prognostic model. The risk score model was constructed using LASSO Cox regression analysis, and nine differentially expressed genes (ESCO2, HAT1, KAT8, HDAC1, HDAC2, HDAC4, HDAC6, SIRT3, and HDAC11) were screened. The risk scoring model had good prediction effectiveness on both TCGA and ICGC datasets. Independent prognostic models suggest that LARs might serve as a potential prognostic prediction in HCC patients, and the high-risk groups exhibits remarkably lower OS rate of HCC patients than the low-risk group. The AUC value of the risk score model in the ICGC and TCGA cohorts performed well in predicting short-term survival (1–3 years). Univariate and multivariate Cox regression analysis of the two cohorts indicated that the prognostic characteristics of LAR-related genes were accurate, reliable, and explainable. GSEA results showed that various tumor-related signal transduction pathways were abundant in the high-risk groups. RT-qPCR results showed that HAT1, HDAC1, HDAC2, HDAC4, and HDAC11 were highly expressed in HCC cells.

We identified nine risk prognostic genes, namely HAT1, KAT8, ESCO2, HDAC1, HDAC2, HDAC4, HDAC6, SIRT3, and HDAC11. The risk score of this feature is related to invasive clinicopathological features and can also function as an independent prognostic factor for overall survival. HAT1 was identified as the first histone acetyltransferase. As a carcinogenic protein, HAT1 may promote cell proliferation and induce cisplatin resistance in HCC. Therefore, targeted HAT1 inhibitors are feasible strategies for the effective treatment of advanced HCC (Jin et al., 2017). KAT8 is mainly involved in the acetylation of histone H4 lysine 16 (H4K16) and certain non-histones. KAT8 consumption significantly promotes HCC cell migration and invasion. (Wei et al., 2021). The role of ESCO2 in HCC has not yet been reported. However, ESCO2 promotes proliferation and metastatic metabolic reprogramming of lung adenocarcinoma cells *in vitro* and *in vivo* (Zhu et al., 2021). Moreover, ESCO2 knockdown inhibits cell proliferation and induces apoptosis in human gastric cancer cells (Chen et al., 2018). HDAC1 and HDAC2 are enzymes that regulate gene transcription and participate in cell cycle progression, differentiation, apoptosis, and tumorigenesis. Pharmacological or transcriptional inhibition of HDAC1 and HDAC2 can lead to cell cycle arrest and apoptosis in HCC (Zhou et al., 2018). Targeted inhibition of HDAC4 has been shown to inhibit the growth and metastasis of HCC, and HDAC4 can promote the proliferation, migration and invasion of nasopharyngeal carcinoma cells *in vitro*, as well as tumor growth and lung metastasis *in vivo* (Freese et al., 2019; Cheng et al., 2021). HDAC6 is a tumor suppressor that inhibits Let-7i-5p to induce TSP1/CD47-mediated anti-tumorigenesis and phagocytosis of HCC. In our study, we found that HDAC6 expression was down-regulated in HCC patients, which is in line with the results of the current study (Yang H. D. et al., 2019). SIRT3 inhibits the growth and cell proliferation and promotes apoptosis in HCC cells. The low expression or lack of SIRT3 in HCC tissues suggests that SIRT3 expression may affect the occurrence and development of HCC (Liu et al., 2021). HDAC11 is the only IV histone deacetylase. It forms a complex with early growth response (Egr-1) of p53 transcription factor, which induces deacetylation of Egr-1, inhibits p53 transcription, and promotes the occurrence of HCC (Gong, 2019).

Tumor microenvironment plays an important role in the occurrence and development of tumor. In many solid organ malignant tumors, tumor infiltrating immune cells have high prognostic value for tumor progression and patient survival (Gajewski et al., 2013; Lamplugh and Fan, 2021). In this study, the proportion of memory-activated CD4 T cells and M0 macrophages was higher in patients with high risk score, which confirmed the role of LARs in the regulation of tumor immune invasion. Functional enrichment analysis showed that LARs was mainly involved in the immune pathway. Therefore, the poor prognosis of high-risk HCC patients may be due to this tumor immunosuppressive microenvironment.

As far as we know, this is the first study to identify the LAR genes related to the prognosis of HCC and to construct the related risk model for prognostication. The model is verified in two external independent HCC queues. LARs features have strong and stable prognostic value and have a broad prospect in clinical application. However, this study still has its limitations. First of all, although our model has been well verified in two large databases, TCGA and ICGC, our study is still a retrospective study, and some prospective studies are needed to verify its clinical application. In addition, it is very important to verify the functional characteristics and molecular mechanism of LARs gene through biological experiments and clinical studies. All in all, further investigations with some wet lab evidence are needed to validate our findings and improve the statistical power to achieve more meaningful results.

## Conclusion

In summary, our results suggest that LARs’ risk score model can be used as a potential prognostic factor of HCC, which may be helpful for personalized management of cancer in the clinical environment.

## Data Availability

The datasets presented in this study can be found in online repositories. The names of the repository/repositories and accession number(s) can be found in the article/Supplementary Material.

## Supplementary Material

Supplementary Figure 1
**(A)** Plots for LASSO expression coefficients of 10 LARs. **(B)** The 2-year alignment diagram calibration curve of the entire TCGA queue. **(C)** The 3-year alignment diagram calibration curve of the entire TCGA queue. **(D)** The 1-year alignment diagram calibration curve of the entire ICGC queue. **(E)** The 2-year alignment diagram calibration curve of the entire ICGC queue. **(F)** The 3-year alignment diagram calibration curve of the entire ICGC queue.

Supplementary Figure 2 Validation of the risk model in the ICGC cohort. **(A)** Survival analysis of patients in the high-risk and low-risk groups based on the prediction risk score formula. **(B)** The median value of risk scores with survival and statuses of HCC patients depends on the risk model in the ICGC cohort and the distribution of risk scores with survival and statuses of HCC patients depends on the risk model in the ICGC cohort. **(C)** One-, two-, and 3-year ROC curves of the risk model for assessing the prognostic performance of the gene signature in the ICGC cohort. **(D)** Principal component analysis of HCC patients in the ICGC cohort. **(E)** t-SNE analysis of HCC patients in the ICGC cohort. **(F–N)** Kaplan–Meier survival analysis of the association between mRNA expression of LARs and OS in HCC patients. The HCC sample information was derived from the ICGC databases.

Supplementary Table S1 Primers used in this study.

Supplementary Table S2 Information of the nine LARs.

## Abbreviations

AUC, area under the curve; ATCC, American Type Culture Collection; CDF, cumulative distribution function; DMEM, Dulbecco’s modified Eagle medium; FBS, fetal bovine serum; GSEA, gene set enrichment analyses; HCC, hepatocellular carcinoma; HDACs, histone deacetylases; HR, hazard ratio; ICGC, International Cancer Genome Consortium; KATs, lysine acetyltransferases; LARs, lysine acetylation regulators; LASSO, least absolute shrinkage and selection operator; OS, overall survival; PTMs, post-translational modification; PPI, protein-protein interaction; PCA, principal component analysis; ROC, receiver operating characteristic; TCGA, The Cancer Genome Atlas; TMB, tumor mutation load; t-SNE, t-distributed stochastic neighbor embedding.
