# Supplementary figures and images for: The Prognostic Value of Lysine Acetylation Regulators in Hepatocellular Carcinoma
Source: Front Mol Biosci. 2022 Mar 9;9:840412. doi: 10.3389/fmolb.2022.840412 (PMC8959434; doi:10.3389/fmolb.2022.840412)

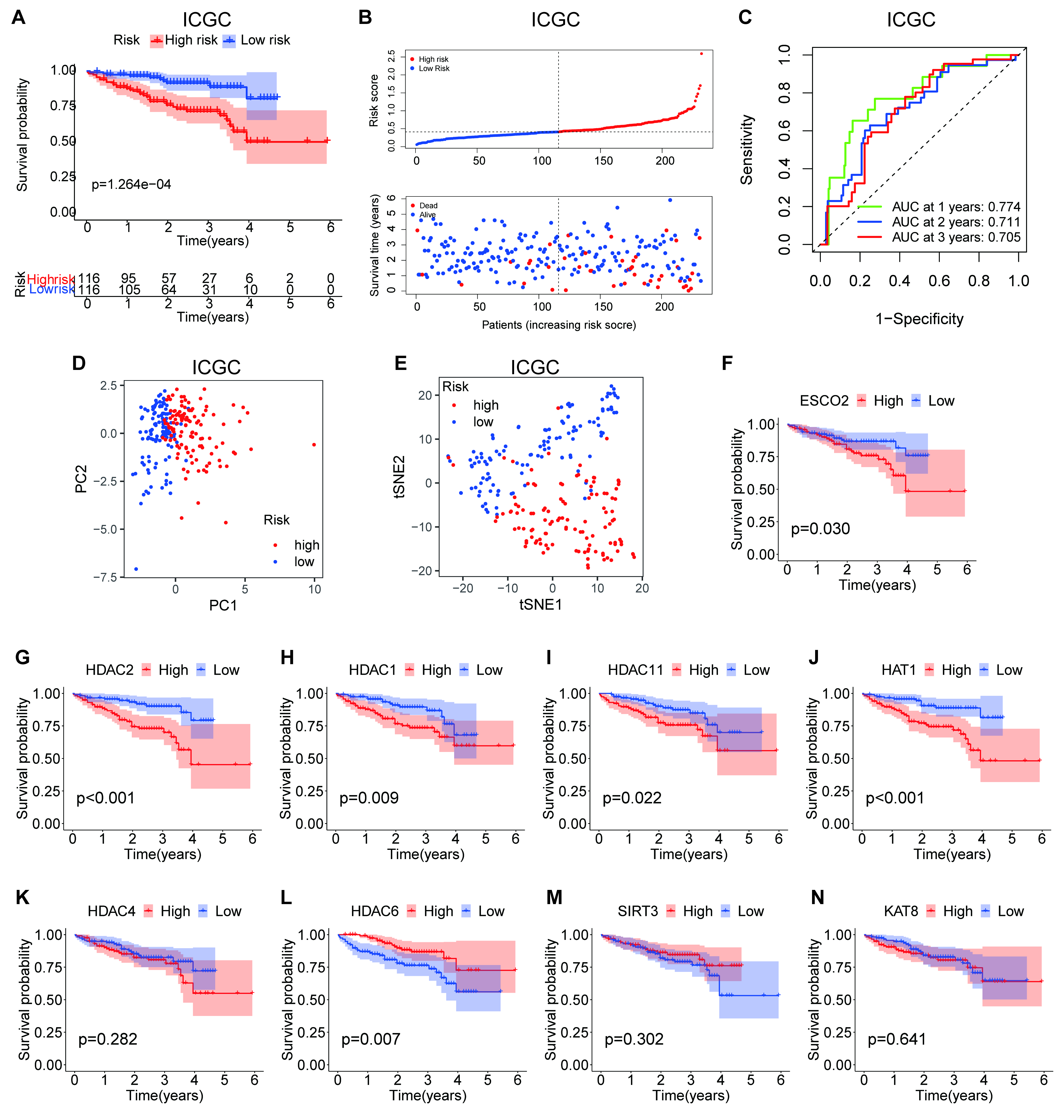

Supplement: Supplementary file 1 [file Image2.TIF]

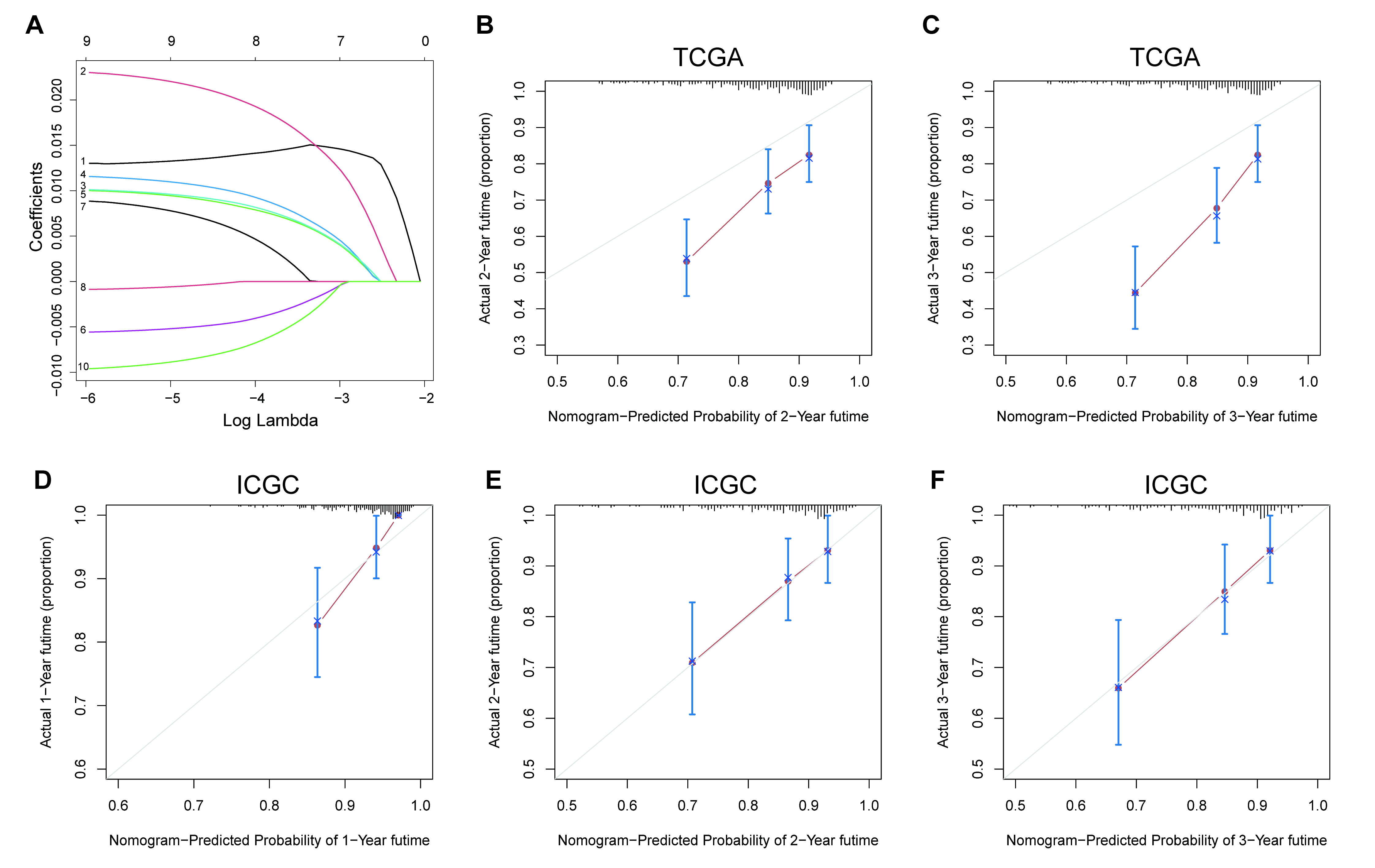

Supplement: Supplementary file 2 [file Image1.TIF]
