# Supplementary material for: The Prognostic Value of Lysine Acetylation Regulators in Hepatocellular Carcinoma
Source: Front Mol Biosci. 2022 Mar 9;9:840412. doi: 10.3389/fmolb.2022.840412 (PMC8959434; doi:10.3389/fmolb.2022.840412)
Supplement: Supplementary file 3 [file DataSheet1.docx]

Supplement Table 1. Information of the 33 LARs.

| **Type** | **Gene** |
| --- | --- |
| **Acetylatransferase** | CREBBP |
|  | EP300 |
|  | ESCO1 |
|  | ESCO2 |
|  | HAT1 |
|  | KAT2A |
|  | KAT2B |
|  | KAT5 |
|  | KAT6A |
|  | KAT6B |
|  | KAT7 |
|  | KAT8 |
|  | SLC16A10 |
| **Deacetylase** | HDAC1 |
|  | HDAC2 |
|  | HDAC3 |
|  | HDAC4 |
|  | HDAC5 |
|  | HDAC6 |
|  | HDAC7 |
|  | HDAC8 |
|  | HDAC9 |
|  | HDAC10 |
|  | HDAC11 |
|  | SIRT1 |
|  | SIRT2 |
|  | SIRT3 |
|  | SIRT4 |
|  | SIRT5 |
|  | SIRT6 |
|  | SIRT7 |
|  | LEF1 |
|  | HNF1A |

Supplement Table 2. Primers used in this study.

| Gene | Direction | Sequences (5’-3’) |
| --- | --- | --- |
| HAT1 | Forward | TGGCGATAGAGGCACAACAG |
|  | Reverse | ACACGCCGGTAATCTTCCAC |
| HDAC1 | Forward | GATTCCTCTCCACACCTGACTTC |
|  | Reverse | TGTTGCCAGAGACGAAGTGGAG |
| HDAC2 | Forward | ATGGCGTACAGTCAAGGAGG |
|  | Reverse | TGCGGATTCTATGAGGCTTCA |
| HDAC4 | Forward | GGCCCACCGGAATCTGAAC |
|  | Reverse | GAACTCTGGTCA AGGGAACTG |
| HDAC11 | Forward | CACGCTCGCCATCAAGTTTC |
|  | Reverse | GAAGTCTCGCTCATGCCCATT |

Supplement Table 3. Information of the nine LARs.

| Gene | Coefficient | Type |
| --- | --- | --- |
| HDAC2 | 0.0135629962458994 | Deacetylase |
| ESCO2 | 0.0211376968068384 | Acetylatransferase |
| HAT1 | 0.00893192417872286 | Acetylatransferase |
| HDAC1 | 0.0104372670066423 | Deacetylase |
| HDAC11 | 0.00915650749552334 | Deacetylase |
| SIRT3 | -0.00486504154762453 | Deacetylase |
| HDAC4 | 0.00679462118339103 | Deacetylase |
| HDAC6 | -0.000338102676486109 | Deacetylase |
| KAT8 | -0.0082215896593953 | Acetylatransferase |
